# Pneumococcal conjugate vaccination schedules in infants—acquisition, immunogenicity, and pneumococcal conjugate and yellow fever vaccine co-administration study: statistical analysis plan

**DOI:** 10.1186/s13063-024-08036-6

**Published:** 2024-03-26

**Authors:** Grant A. Mackenzie, Isaac Osei, Rasheed Salaudeen, Paul V. Licciardi, Brian Greenwood, Kim Mulholland, Cattram Nguyen

**Affiliations:** 1 MRC Unit The Gambia at London School of Hygiene & Tropical Medicine, Fajara, The Gambia; 2 Faculty of Infectious and Tropical Diseases, London School of Hygiene & Tropical Medicine, London, UK; 3 Infection and Immunity Theme, Murdoch Children’s Research Institute, Melbourne, Australia; 4 Department of Paediatrics, University of Melbourne, Melbourne, Australia; 5 Faculty of Epidemiology and Public Health, London School of Hygiene & Tropical Medicine, London, UK

**Keywords:** Pneumococcal, Vaccine, Schedule, Cluster-randomised controlled trial, Statistical analysis, Immunogenicity, Acquisition

## Abstract

**Rationale:**

The effectiveness of immunisation with pneumococcal conjugate vaccine (PCV) has been demonstrated in many countries. However, the global impact of PCV is limited by its cost, which has prevented its introduction in some countries. Reducing the cost of PCV programmes will facilitate further vaccine introductions and improve the sustainability of PCV in low-income countries when they transition from subsidised vaccine supply. We are conducting a large, population-level, cluster-randomised field trial (PVS) of an alternative reduced-dose schedule of PCV compared to the standard schedule. We are also conducting a nested sub-study at the individual level to investigate the immunogenicity of the two schedules and their effects on pneumococcal carriage acquisition (PVS-AcqImm).

**Methods and design:**

PVS-AcqImm is a prospective, cluster-randomised trial of an alternative schedule of one dose of PCV scheduled at age 6 weeks with a booster dose at age 9 months compared to the standard of three primary doses scheduled at 6, 10, and 14 weeks of age. Sub-groups within the alternative schedule group receive yellow fever vaccine separately or co-administered with PCV at 9 months of age. The primary endpoints are (a) concentrations of vaccine-type anti-pneumococcal IgG at 18 months of age, (b) proportions with yellow fever neutralising antibody titre ≥ 1:8 4 weeks after separate or co-administration of PCV and yellow fever vaccines, and (c) rate of nasopharyngeal vaccine-type pneumococcal acquisition from 10–14 months of age. Participants and field staff are not masked to group allocation while measurement of the laboratory endpoints is masked. Approximately equal numbers of participants are resident in each of 28 randomly allocated geographic clusters (14 clusters in each group); 784 enrolled for acquisition measurements and 336 for immunogenicity measurements.

**Purpose:**

This statistical analysis plan (SAP) describes the PVS-AcqImm cohort and follow-up criteria to be used in different analyses. The SAP defines the endpoints and describes how adherence to the interventions will be presented. We describe the approach to analyses and how we will account for the effect of clustering. Defining the SAP prior to the conduct of analysis will avoid bias in analyses that may arise from prior knowledge of trial findings.

**Trial registration:**

ISRCTN, ISRCTN7282161328. Registered on 28 November 2019. https://www.isrctn.com/ISRCTN72821613.

Protocol: MRCG SCC number 1670, LSHTM Ref 17683.

Current protocol version: 6.0, 24 May 2021.

Version: 1.0 (5 April 2023); SAP revisions—none.

**Supplementary Information:**

The online version contains supplementary material available at 10.1186/s13063-024-08036-6.

## Introduction

This statistical analysis plan (SAP) follows the guidelines described by Rodriguez et al [1] and the UK Clinical Research Collaboration Registered Clinical Trial Unit Statisticians’ Operational Group [2], as well as CONSORT guidelines for cluster-randomised trials [3]. The trial protocol is available on the *Trials*website [4].

### Background and rationale

The Gambian Expanded Programme on Immunisation (EPI) introduced 7-valent pneumococcal conjugate vaccine (PCV7) in 2009, and PCV13 in 2011, using the standard schedule of three doses in early infancy. Compared to the pre-vaccine period, in 2016–17, there was a 92% reduction in the incidence of vaccine-type (VT) invasive pneumococcal disease (IPD) in the 2–59-month age group [5]. A major effect of PCV relates to its prevention of acquisition of VT pneumococcal carriage and transmission, with resulting indirect herd protection effects at the population-level. However, the prevalence of nasopharyngeal (NP) carriage of VT pneumococci in young Gambia children remains around 15% (author’s own data), having fallen from 47% in the pre-vaccine period [6]. The global impact of PCV is limited by its cost, which has prevented its introduction in some countries. Reducing the cost of PCV programmes may facilitate vaccine introduction in such countries and improve the sustainability of EPI programmes in low-income countries when they transition away from subsidised vaccine supply through Gavi, the Vaccine Alliance. This trial is part of a global initiative to generate evidence about reduced dose schedules for PCV. The PVS-AcqImm trial aims to measure individual-level effects of a reduced dose schedule compared to the standard schedule, which will complement the larger PVS field trial with its population-level endpoints.

Several studies indicate that immunological priming for an optimal PCV booster dose response may be more effective with fewer primary doses [7, 8]. In addition, the immunological response to a booster dose following a single priming dose may reduce VT acquisition to a greater degree than following the standard 3 + 0 schedule [9]. As a result, a schedule with one primary dose and a later booster dose may induce greater herd protection than the standard schedule. The duration of protection of PCV is poorly defined but the potential for greater antibody persistence following a booster dose compared to doses in early infancy [10, 11] suggests that protection may be more long-lived when a booster dose is given [12]. The relative effect of a PCV booster dose on the density of VT carriage is also unclear but such an effect may influence the risk of transmission. Immunological correlates of effect on NP acquisition are also poorly defined but would be valuable for the evaluation of new PCVs. A potential barrier to the use of booster doses of PCV in low-income countries is the lack of evidence of safe co-administration with yellow fever (YF) vaccine, which in Africa is generally scheduled at 9 months of age.

The PVS-AcqImm sub-study aims to conduct an individual-level immunogenicity and acquisition study within the larger PVS field trial. Immunogenicity data will be important to interpret the PVS results. Immunogenicity data on the reduced dose schedule are needed in a typical African population given that administration of the first dose is likely to be earlier than in developed countries, concentrations of maternally derived antibody, which may affect responses to the primary dose(s), are different in populations with high pneumococcal transmission, and responses to PCV may be quantitatively different in this population compared to others. Similarly, empiric measurement of the effect of the PCV13 booster dose on VT acquisition will provide direct evidence of the relative effect of the two schedules on herd protection and assist interpretation of the PVS results. We will also generate evidence about immunological responses when PCV and YF are co-administered. These data will assist policy-makers in consideration of reduced dose PCV schedules to reduce costs and increase the flexibility, acceptability, and sustainability of EPI programmes.

### Objectives

PVS-AcqImm aims to compare pneumococcal acquisition and immunogenicity in individual infants receiving an alternative schedule of PCV13 with doses scheduled at 6 weeks and 9 months of age compared to standard scheduling of doses at 6, 10, and 14 weeks of age. We will also determine if co-administration of PCV13 and YF vaccine is associated with non-inferior immune responses.

#### Endpoints

The primary endpoints are as follows:

- Pneumococcal VT serotype-specific IgG concentration at 18 months of age.
- Proportion of participants with protective titres of YF neutralising antibodies after separate or co-administration of PCV and YF vaccines.
- Rate of NP pneumococcal VT acquisition from 10 to 14 months of age.

The secondary endpoints are as follows:

- Rate of NP acquisition of non-VT pneumococci from 10 to 14 months of age.
- Proportion of participants with NP VT colonisation at 6, 9, and 18 months of age.
- Proportion of participants with pneumococcal VT IgG concentration ≥ 0.35 µg/ml, 4 weeks after the primary series, i.e. 4 weeks after the first dose in the alternative schedule and 4 weeks after the third dose in the standard schedule, 4 weeks after the booster dose at age 9 months, i.e. at 10 months of age in both groups, and at 18 months of age.
- Pneumococcal VT opsonophagocytic activity (OPA) following a single dose at age 6 weeks in the alternative schedule group, following three primary doses in the standard schedule group, following the booster at age 9 months, i.e. at 10 months of age in both groups, and at 18 months of age.
- Geometric mean concentrations (GMC) of pneumococcal VT IgGs 4 weeks after administration of PCV13 at 9 months of age with and without co-administration with YF vaccine.

#### Other analyses

The most recent amendment to the trial protocol includes extended follow-up and measurement of additional endpoints:

- Rate of NP acquisition of VT pneumococci from 23–28 months of age.
- Rate of NP acquisition of non-VT pneumococci from 23–28 months of age.
- Density of VT pneumococcal colonisation at 10 months of age.
- Correlation of serotype-specific IgG and OPA at age 10 months and rate of homologous serotype acquisition between 10 and 14 months of age.
- Correlation of serotype-specific IgG and OPA at age 18 months and rate of homologous serotype acquisition between 23 and 28 months of age.

## Study methods

### Trial design

PVS-AcqImm is a parallel group, unmasked, cluster-randomised trial of the individual-level effect of two different schedules of PCV13. This trial is nested within the PVS field trial but designed for interpretation of effects at the individual-level. This is a phase IV trial involving licenced products comparing alternative and standard schedules for PCV13 and separate versus co-administration with YF vaccine. Participants are resident in population clusters which were allocated to the two groups in a 1:1 ratio.

#### Definition of cluster and design application to clusters

The trial area in the Basse Health and Demographic Surveillance System (BHDSS) consists of 28 contiguous geographic units within which resident infants are assigned to attend the one EPI clinic in that geographic area [4]. Group allocation is determined by the group allocation of the village of residence.

#### Intervention

Infants resident in PVS-AcqImm clusters and aged 0–10 weeks are allocated to receive either the alternative or standard PCV schedule. The alternative schedule specifies eligibility for doses at 6 weeks and 9 months of age. The standard schedule specifies eligibility for doses at 6, 10, and 14 weeks of age. The participants in each alternative schedule cluster who are assigned to the measurement of immunogenicity endpoints are further assigned to receive the YF vaccine co-administered at 9 months of age with the PCV booster dose or administered separately at 10 months of age (Table 1).

**Table 1:** Trial groups and schedule of vaccination, specimen collection, and endpoint measurement

	Group		
Age	3 + 0 Acquisition (*n* = 392) Immunogenicity (*n* = 112)	1 + 1 Acquisition (*n* = 196) Immunogenicity—YF/PCV co-administration (*n* = 112)	1 + 1 YF_separate_ Acquisition (*n* = 196) Immunogenicity—YF/PCV separate administration (*n* = 112)
6 weeks	*PCV13* *NPS* (*n* = 392)	*PCV13* *NPS* (*n* = 196)	*PCV13* *NPS* (*n* = 196)
10 weeks	*PCV13*	*Blood* IgG (*n* = 112) OPA (*n* = 60)	
14 weeks	*PCV13*		
18 weeks	*Blood* IgG (*n* = 112) OPA (*n* = 60)		
6 months	*NPS* (*n* = 392)	*NPS* (*n* = 196)	*NPS* (*n* = 196)
9 months	*YF* *Blood* IgG (*n* = 112) *NPS* (*n* = 392)	*YF and PCV13* *Blood* IgG (*n* = 56) *NPS* (*n* = 196)	*PCV13* *Blood* IgG (*n* = 56) *NPS* (*n* = 196)
10 months	*Blood* YFNA (*n* = 112) IgG (*n* = 112) OPA (*n* = 112) *NPS and serotype-specific carriage density* (*n* = 392)	*Blood* YFNA (*n* = 112) IgG (*n* = 112) OPA (*n* = 112) *NPS and serotype-specific carriage density* (*n* = 196)	*YF* *Blood* IgG (*n* = 112) *NPS and serotype-specific carriage density* (*n* = 196)
11 months	*NPS* (*n* = 392)	*NPS* (*n* = 196)	*Blood* YFNA (*n* = 112) *NPS* (*n* = 196)
12 months	*NPS* (*n* = 392)	*NPS* (*n* = 196)	*NPS* (*n* = 196)
13 months	*NPS* (*n* = 392)	*NPS* (*n* = 196)	*NPS* (*n* = 196)
14 months	*NPS* (*n* = 392)	*NPS* (*n* = 196)	*NPS* (*n* = 196)
18 months	*Blood* IgG (*n* = 112) OPA (*n* = 112) *NPS* (*n* = 392)	*Blood* IgG (*n* = 112) OPA (*n* = 112) *NPS* (*n* = 196)	*NPS* (*n* = 196)
23 months	*NPS* (*n* = 392)	*NPS* (*n* = 196)	*NPS* (*n* = 196)
24 months	*NPS* (*n* = 392)	*NPS* (*n* = 196)	*NPS* (*n* = 196)
25 months	*NPS* (*n* = 392)	*NPS* (*n* = 196)	*NPS* (*n* = 196)
26 months	*NPS* (*n* = 392)	*NPS* (*n* = 196)	*NPS* (*n* = 196)
27 months	*NPS* (*n* = 392)	*NPS* (*n* = 196)	*NPS* (*n* = 196)
28 months	*NPS* (*n* = 392)	*NPS* (*n* = 196)	*NPS* (*n* = 196)

### Randomisation

An independent statistician prepared the randomisation lists. All clusters were randomised at the beginning of the PVS trial using a blocked scheme to ensure similar numbers of clusters were assigned to each group. Randomisation was stratified by a binary variable of “high” or “low” cluster-level incidence of clinical pneumonia. Randomisation was carried out in permutations until restricted selections achieved balance in terms of the presence of a health facility in allocated clusters, i.e. two health facilities in each group. Random allocation of the clusters was performed and revealed in a public event in which one of 100 valid randomisation lists was randomly selected. We used pre-prepared random lists to allocate participants to the PVS-AcqImm sub-groups for measurement of immunogenicity endpoints, including PCV13-YF vaccine co-administration, and NP carriage acquisition alone (Table 1). Consent is sought at EPI clinics to receive the interventions and follow-up specific to PVS-AcqImm and the main PVS trial [4, 13].

### Statistical considerations and sample size

#### Effect sizes

For measurement of the effect of the PCV booster dose on the rate of acquisition of VT pneumococci, the smallest clinically important hazard ratio to detect was set at 0.75. This is a more conservative value than that observed in a Dutch study of VT colonisation following a PCV booster at 12 months of age compared to no booster, which showed a relative risk reduction of 0.64 [9].

Serotype-specific antipneumococcal IgG to the serotypes included in PCV13 will be analysed as a fold increase in GMCs as our interest is to test whether antibody concentrations are different and not necessarily correlates of protection. The smallest effect size of clinical significance is a 1.8-fold or greater increase in serotype-specific GMC, an approach used in other similar studies [14]. The 1.8-fold increase is based on data comparing IgG concentrations in Vietnamese children receiving PCV10 schedules of 3 + 1 or 3 + 0 in whom GMCs 1 month following the booster dose were threefold or more greater for all vaccine types (pers. comm. P Licciardi 23 Apr 2018). The overall hypothesis is based on 13 tests of the null hypothesis of no difference in serotype-specific GMC. That is, overall superiority will be declared if at least 10/13 hypothesis tests are rejected. Non-inferiority of response to YF vaccine will be defined as the lower limit of the 95% confidence interval for the absolute difference in proportions with neutralising titres of YF antibody ≥ 1.8 being greater than − 10% [15, 16].

#### Sample size calculation

PVS-AcqImm includes two primary immunogenicity endpoints and sample size calculations for each used *α* = 0.025 and *β* = 0.9. For the first primary endpoint, our interest is in the relative magnitude of the serotype-specific IgG response in the two groups. The second primary endpoint is the proportion of participants with protective YF seroresponses in separate and co-administration groups. The effect of clustering was taken into account using an ICC = 0.01 based on assumptions using local data. The sample size to determine differences in rates of serotype-specific NP acquisition was calculated separately assuming *α* = 0.025 and *β*= 0.9. A full description of the sample size considerations is found in the protocol [4].

### Framework

The hypothesis framework is superiority of the alternative versus standard schedule to reduce NP acquisition of VT pneumococci from 10–14 to 23–28 months of age and VT immune response at age 18 months, with inference at the individual level. We will test the non-inferiority of the immune response following co-administration versus separate administration of PCV and YF vaccines. We will test the superiority of the alternative schedule to reduce VT carriage density at age 10 months.

### Interim analyses and stopping guidance

#### Interim analyses

No interim analyses are planned.

#### Guidance for stopping the trial early

As with PVS, PVS-AcqImm may be stopped early if there is evidence that the risk of pneumococcal disease is greater in one compared to the other group. Rather than specify a formal stopping rule, the Data Monitoring Committee (DMC) regularly reviews the accruing data. If the DMC recommends that the trial be stopped early, a joint meeting of the DMC, Trial Steering Committee, and Central Stakeholder Committee will make a recommendation to the Sponsor regarding post trial procedures.

### Timing of analyses

The first phase of analysis will be conducted when data are complete for follow-up to 18 months of age, including all three primary endpoints. A second phase of analysis will include complete follow-up data to 28 months of age.

### Timing of outcome assessments

#### Timing of primary endpoint assessment

The primary outcome of NP VT acquisition is measured between 10 and 14 months of age. The primary endpoint of serotype-specific IgG concentration is measured at 18 months of age. The primary endpoint of YF vaccine response is measured 1 month post YF vaccination in the co-administration and separate administration groups.

#### Timing of secondary endpoint assessments

Secondary endpoints of VT pneumococcal carriage prevalence are measured at 6, 9, and 18 months of age, VT acquisition at 23–28 months of age, and non-VT (NVT) acquisition at 10–14 and 23–28 months of age. Serotype-specific pneumococcal IgG concentration and OPA are measured 4 weeks after the primary series of PCV, around 10 months of age, i.e. 1 month post booster in the alternative schedule group, and at 18 months of age. The density of VT pneumococcal colonisation is measured around 10 months of age, i.e. 1 month post booster in the alternative schedule group. Serotype-specific NP acquisition between 10–14 and 23–28 months of age will be correlated with serotype-specific IgG concentration and OPA at 10 and 18 months of age, respectively.

## Statistical principles

### Confidence intervals and *p*-values

#### Level of statistical significance

The level of statistical significance for all hypothesis tests will be an α-level of 0.05 using two-sided significance tests [17, 18]. We specify two-sided *α*-levels of 0.05 as we wish to know the lower, as well as upper limit of the confidence interval around the effect estimates, as it is plausible that the alternative schedule may be superior to the standard schedule.

#### Multiplicity

To account for inflated type I error due to testing 13 different serotypes, we have defined an overall hypothesis based on the rejection of ≥ 10/13 null hypotheses. Study power for the primary endpoint of serotype-specific IgG GMCs is 99%. No further adjustment will be made to the level of significance for multiple hypothesis tests. Hypothesis tests will be presented with specific *p*-values for researchers to interpret within the context of all presented results and all the available evidence in the field [19].

#### Confidence intervals

Two-sided 95% confidence intervals will be used for all superiority and non-inferiority endpoints [17, 18]. To account for the effect of clustering and stratification in the randomisation, statistical models will account for cluster and include stratification variables as covariates.

### Adherence and protocol deviations

#### Definition and assessment of adherence to the intervention

##### Definitions of adherence

Vaccination per-protocol (PP) will be defined as a child enrolled into the study at ≤ 70 days (≤ 10 weeks) of age and who received the allocated schedule of PCV doses during follow-up. PP administration of the standard schedule will require administration of the first dose of PCV at ≤ 84 days (≤ 12 weeks) of age and the third dose at ≤ 154 days (≤ 22 weeks) of age. PP administration of the alternative schedule will require administration of the first dose at ≤ 84 days (≤ 12 weeks) of age and the second dose at ≥ 273 days (≥ 9 months) and ≤ 350 days (≤ 11.5 months) of age [4]. PP administration of YF vaccine in the co-administration group will require receipt at ≥ 273 days (≥ 9 months) and ≤ 350 days (≤ 11.5 months) of age with PCV administered on the same day. PP administration of YF vaccine in the separate administration group will require PCV administration ≥ 28 days prior to YF vaccine, which will be administered at ≥ 304 days (≥ 10 months) and ≤ 380 days (≤ 12.5 months) of age [4]. Participants receiving vaccines outside these criteria will be included in intention-to-treat (ITT) analyses, in groups as randomised.

Incomplete vaccination in both groups will be defined as administration of zero or one dose of PCV and administration of two doses in the standard schedule group.

Participants resident in a village allocated to the standard schedule will be defined as cross-over between groups if doses of PCV are received at an age when a dose will function as a booster, as is the intention of the alternative schedule. For infants allocated to the standard schedule, cross-over will be defined as administration of (a) two doses of PCV with the second dose administered at ≥ 252 days (≥ 36 weeks) of age with an interval of > 152 days (> 21 weeks) or (b) three doses of PCV with the third dose administered at ≥ 252 days (≥ 36 weeks) of age with an interval of > 152 days (> 21 weeks) between the first and third doses [20]. The definition of cross-over for infants resident in villages allocated to the alternative schedule is related to receiving three or more doses of PCV at an age when doses will not function as a booster, as is the intention of the standard schedule. For infants allocated to the alternative schedule, cross-over is defined as administration of (a) three doses of PCV with administration of the third dose < 252 days (< 36 weeks) of age or (b) four doses with administration of the fourth dose < 252 days (< 36 weeks) of age. Infants that migrate internally between clusters allocated to different schedules, before completing their PCV schedule, may validly change group allocation to that of the destination cluster, but migrations after completing their PCV schedule will be classified as cross-overs from the date of migration.

Vaccination status will be defined as out-migration or death before completion if the child migrates out or dies stat an age before which their PCV schedule is completed. Infants in the standard schedule group who out-migrate or die ≤ 112 days (≤ 16 weeks) of age and receive zero doses of PCV will be classified as ‘out-migration/death, unvaccinated before age 16 weeks’ and ‘out-migration/death, incomplete before age 16 weeks’ if one or two doses are received. Infants in the alternative schedule group who out-migrate or die before age ≤ 294 days (≤ 42 weeks) and receive zero doses of PCV will be classified as ‘out-migration/death, unvaccinated before age 42 weeks’ and ‘out-migration/death, incomplete before age 42 weeks’ if one dose of PCV is received.

##### Assessment of adherence

Adherence will be assessed, by group, as (a) PP vaccination, (b) incomplete vaccination, (c) cross-over, or (d) migration/death before completion, in the cohort at 548 days (18 months) of age, i.e. during follow-up for the primary endpoint analyses, and at 852 days (28 months) of age, i.e. during follow-up for the endpoints measured at that age.

#### Presentation of adherence to the intervention

Adherence among participants will be presented in tabular and graphical forms. A frequency table will show the proportions in each group who fall into the aforementioned categories of adherence. Histograms with stacked 100% bars will represent the proportions of participants at 18 months and 28 months of age in each group, in each category of adherence.

#### Definition of protocol deviations

Definitions of protocol deviations are given in Table 2.

**Table 2:** Definition of protocol deviations

Reason for protocol deviation	
Enrolment of ineligible participant	See Sect. 4.2 Eligibility
**Non-adherence to vaccine schedule**	
Incorrect dose given	1st dose—age < 6 weeks
	Standard schedule—received > 3 doses
	Standard schedule—3rd dose age > 22 weeks
	Alternative schedule—2nd dose age < 40 weeks
	Alternative schedule—received > 2 doses
Withdrawn, received alternative schedule	Withdrawn—2nd dose age ≥ 36 weeks
**Non-adherence to endpoint timeline**	
Blood	Collection > 35 days from specified date
NP specimen	< 14 days between consecutive specimens
	Collection at 16 or 17 months of age (standard schedule)
	Collection > 6 months after booster administration and < 18 months of age (alternative schedule)
	18-month specimen collection > 20 months of age
	28-month specimen collection > 30 months of age

#### Which protocol deviations will be summarised

Protocol deviations will be summarised as in Table 2.

### Analysis cohorts

The PP and ITT cohorts will include participants who meet the criteria set out in Sect. 3.2.1. Analyses of endpoints measured at different ages will include cohorts that meet the specified criteria during follow-up to the specified age and if there is a laboratory result. For example, the cohort included in the analysis of an endpoint measured at 1 month post booster may be different to the cohort included for an endpoint measured at 18 months of age. Participants classified as incomplete vaccination status, cross-over, out-migration, death, or withdrawal at each endpoint measurement time point will be excluded from PP analyses. All endpoints will be analysed in both PP and ITT cohorts.

## Trial population

### Screening

The trial is being conducted in 28 geographical clusters the BHDSS in rural Gambia. The sampling frame is all infants resident in the 28 clusters and attending EPI clinics. We will describe the numbers and characteristics of resident infants aged 0–52 weeks, born in the trial area, who (a) never present to EPI clinics, (b) present only once to an EPI clinic, and (c) present on multiple occasions to EPI clinics. Characteristics will include sex, mother’s age at the child’s birth, number of household members, number of household children aged < 15 years, ethnicity, and mortality. Thus, we will evaluate the representativeness of the trial sample to the source population.

Screening was implemented by selecting all infants presenting to EPI clinics in each cluster until the target of 28 participants were enrolled in each cluster. More or less than 28 participants per cluster were enrolled, as per protocol, to account for some ‘slow to recruit’ clusters (see Sect. 4.3).

### Eligibility

Participants must be resident in the 28 selected clusters and aged 0–10 weeks. Infants were excluded if:

- Aged ≥ 11 weeks
- Prematurity < 34 weeks gestation
- Weight < 2.5 kg
- History of invasive bacterial infection or measles
- Receiving antibiotics therapy > 4 weeks
- HIV infection in the infant or mother
- Chronic debilitating condition
- Immunosuppressive therapy or immunodeficiency disorder
- Contraindication to PCV13 or YF vaccine

### Recruitment

A target of 28 infants in each of the 28 clusters have been enrolled in PVS-AcqImm, i.e. a target of 784 participants for measurement of pneumococcal acquisition (Table 1). If 28 infants could not be enrolled in ‘slow to recruit’ clusters, enrolments were redistributed to other clusters, resulting in some clusters having less or more than 28 participants. Of the target 392 participants in the alternative schedule group, a target subsample of 224 were enrolled for measurement of immunogenicity endpoints (Table 1), aiming for 16 in each of the 14 clusters. Of the 16 infants targeted in each alternative schedule cluster, a target of eight were allocated to co-administration of PCV and YF vaccine and a target of eight to separate administration, i.e. a target of 112 infants were allocated to separate and co-administration groups (Table 1). Of the target 392 participants in the standard schedule group, a target subsample of 112 were enrolled for measurement of immunogenicity endpoints (Table 1).

**Fig. 1:**
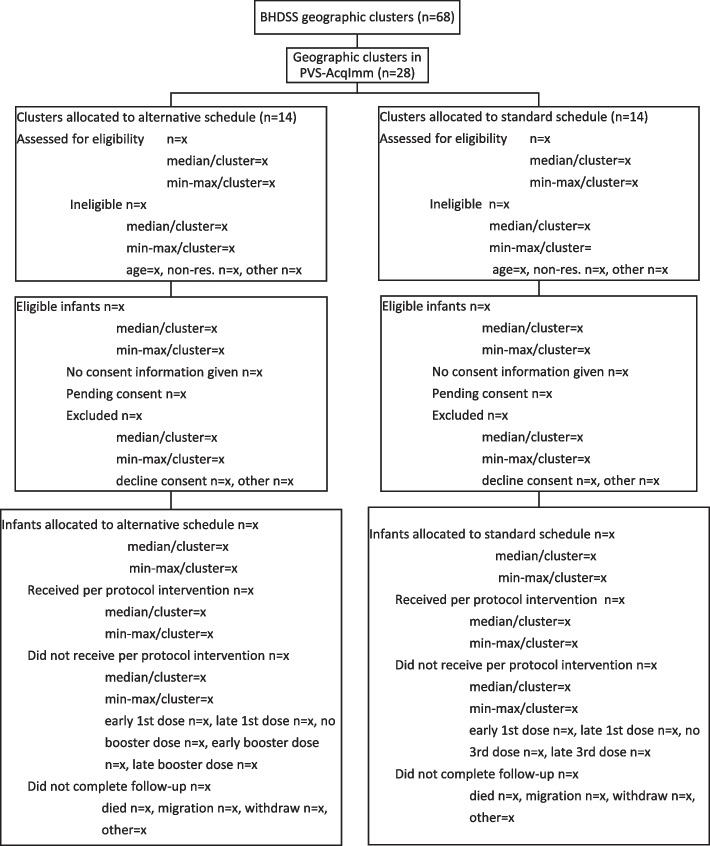
CONSORT diagram of infant screening, eligibility, and enrolment

Enrolment in PVS-AcqImm proceeded over a period of 12 months. Allocation of the subsample of infants to immunogenicity measurements and the PCV-YF vaccine separate or co-administration groups followed a pre-prepared random allocation list for each cluster. If participants withdrew or were lost to follow-up, replacement enrolments occurred in both groups to ensure sufficient enrolment and identical procedures in the two groups. Information to include in the CONSORT flow diagram of enrolment is illustrated in Fig. 1.

### Withdrawal/follow-up

Participants who withdrew from receipt of the alternative schedule do not continue follow-up of endpoint measurement but we continue to record vaccine administration. Participants who die or migrate out of the trial area are lost to follow-up. Data accrued prior to loss to follow-up will be retained for analysis.

### Baseline characteristics

#### Descriptive summary of participant baseline characteristics

The characteristics of participants enrolled to measure acquisition endpoints will be summarised by group, as in Table 3. The characteristics of participants enrolled to measure immunogenicity endpoints will be summarised as in Supplementary Table 1. Participant characteristics will also be summarised at the cluster-level (Supplementary Table 2) [3].

**Table 3:** Baseline characteristics of participants enrolled to measure carriage endpoints

Characteristics	Group	
	***X***	***Y***
No. enrolled	*n* = *x*	*n* = *y*
Age at enrolment (days), *n*	*n* = *x*	*n* = *y*
Median (IQR)	*x* (*x* − *x*)	*y* (*y* − *y*)
Sex, *n*	*n* = *x*	*n* = *y*
Female, *n* (%)	*x* (*x.x*%)	*y* (*y.y*%)
Mother's age (years), *n*	*n* = *x*	*n* = *y*
Median (IQR)	*x* (*x* − *x*)	*y* (*y* − *y*)
Gestational age at birth, *n*	*n* = *x*	*n* = *y*
Median (IQR)	*x* (*x* − *x*)	*y* (*y* − *y*)
Birth weight, *n*	*n* = *x*	*n* = *y*
Median (IQR)	*x* (*x* − *x*)	*y* (*y* − *y*)
Breast fed at enrolment, *n*	*n* = *x*	*n* = *y*
Yes, *n* (%)	*x* (*x.x*%)	*y* (*y.y*%)
Age at first PCV dose (days), *n*	*n* = *x*	*n* = *y*
Median (IQR)	*x* (*x* − *x*)	*y* (*y* − *y*)
Age at second PCV dose (days), *n*	*n* = *x*	*n* = *y*
Median (IQR)	*x* (*x* − *x*)	*y* (*y* − *y*)
Age at third PCV dose (days), *n*	*n* = *x*	*n* = *y*
Median (IQR)	*x* (*x* − *x*)	*y* (*y* − *y*)
Age at PCV booster (alternative schedule) or measles vaccine (standard schedule) [days], *n*	*n* = *x*	*n* = *y*
Median (IQR)	*x* (*x* − *x*)	*y* (*y* − *y*)
Antibiotics since birth, *n*	*n* = *x*	*n* = *y*
Yes, *n* (%)	*x* (*x.x*%)	*y* (*y.y*%)
Smoker in house, *n*	*n* = *x*	*n* = *y*
Yes, *n* (%)	*x* (*x.x*%)	*y* (*y.y*%)
Household cooking fuel, *n*	*n* = *x*	*n* = *y*
Wood, *n* (%)	*x* (*x.x*%)	*y* (*y.y*%)
Charcoal, *n* (%)	*x* (*x.x*%)	*y* (*y.y*%)
Gas, *n* (%)	*x* (*x.x*%)	*y* (*y.y*%)
Electricity, *n* (%)	*x* (*x*.*x*%)	*y* (*y*.*y*%)
Dung, *n* (%)	*x* (*x.x*%)	*y* (*y.y*%)
Infant in cooking area daily, *n*	*n* = *x*	*n* = *y*
Yes, *n* (%)	*x* (*x.x*%)	*y* (*y.y*%)
No. household children aged < 15 years, *n*	*n* = *x*	*n* = *y*
Median (IQR)	*x* (*x* − *x*)	*y* (*y* − *y*)
Age at first NP specimen post PCV booster (alternative schedule) or post measles vaccine (standard schedule) [days], *n*	*n* = *x*	*n* = *y*
Median (IQR)	*x* (*x* − *x*)	*y* (*y* − *y*)
No. NP specimens from 10 to 14 months of age, *n*	*n* = *x*	*n* = *y*
Median (IQR)	*x* (*x* − *x*)	*y* (*y* − *y*)
Age at 18-month blood specimen (days), *n*	*n* = *x*	*n* = *y*
Median (IQR)	*x* (*x* − *x*)	*y* (*y* − *y*)
Age at post booster/post YF vaccine blood specimen (days), *n*	*n* = *x*	*n* = *y*
Median (IQR)	*x* (*x* − *x*)	*y* (*y* − *y*)

#### Descriptive analysis of intervention delivery

Descriptive analysis of the delivery of the intervention to individual participants will be performed as described Sect. 3.2.2.

#### Descriptive analysis of clinical events

We will describe the number and proportion of participants in each group who were detected with hospital admission, clinical pneumonia, radiological pneumonia, IPD, and mortality.

## Analysis

### Outcome definitions

#### Pneumococcal carriage and acquisition

Pneumococcal VT carriage will be defined as detection of pneumococcus in a NP swab including serotypes 1, 3, 4, 5, 6A, 6B, 7F, 9 V, 14, 18C, 19A, 19F, and 23F. Cross-reactive serotype 6C will be defined as a VT [21, 22].

All other serotypes will be defined as NVT. Non-typeable serotypes will be excluded. An event of pneumococcal acquisition will be defined as detection by latex sweep serotyping of a pneumococcal serotype that was not detected in the previous NP specimen. Participants will be included in acquisition analyses if three or more of the scheduled NP specimens are collected between 10–14 and 23–28 months of age. Counts of acquisition may include multiple acquisitions of the same serotype. The primary outcome will be the cumulative proportion of participants with VT acquisition between 10 and 14 months of age [23]. Secondary outcomes will be cumulative VT acquisition between 23 and 28 months of age, mean counts of VT acquisition between 10–14 and 23–28 months age, proportions with VT carriage at baseline, 6, 9, 18, and 24 months of age, as well as the aforementioned outcomes but for NVTs.

#### Pneumococcal IgG

Serotype-specific antipneumococcal IgG concentrations will be reported in units of μg/ml and compared between groups as GMCs and proportions of participants with IgG concentration ≥ 0.35 μg/ml. The primary outcome will be compared as ratios of serotype-specific GMCs for VTs at 18 months of age. Secondary outcomes will be serotype-specific GMCs for VTs and proportions with VT IgG concentrations ≥ 0.35 µg/ml at 1 month post primary series, i.e. 1 month after the first dose in the alternative schedule and 1 month after the third dose in the standard schedule, serotype-specific GMCs, GMC ratios, and proportions with VT IgG concentrations ≥ 0.35 μg/ml at 9 months of age, 1 month post booster, at 18 months of age, and 1 month post YF vaccination in the PCV-YF vaccine separate versus co-administration groups.

#### Pneumococcal opsonophagocytic activity

Serotype-specific antipneumococcal OPA will be reported as an opsonisation index and geometric mean opsonisation indices. Secondary outcomes will be opsonisation indices and geometric means for VTs 1 month post primary series, 1 month post booster, and at 18 months of age.

#### Yellow fever vaccine seroresponse

The seroresponse to YF vaccine will be reported as neutralisation titres that express the serum dilution that yields ≥ 50% neutralisation of cellular infections, i.e. in which one or both of two duplicate infections are blocked. Seroprotection is defined as a neutralisation titre ≥ 1:8. The primary outcome will be proportions with YF neutralisation titres ≥ 1:8, 1 month after administration of YF vaccine.

#### Pneumococcal carriage density

Pneumococcal carriage density will be defined as detection and quantification of pneumococcal genome density as copy number per millilitres, using lytA qPCR methods. The relative abundance of all pneumococcal serotypes present in lytA positive samples will be determined using an established serotype-specific pneumococcal microarray. Serotype-specific carriage density will be determined as log-transformed pneumococcal gene copies per ml multiplied by the relative abundance of each serotype. A secondary outcome will be the aggregate density of all VT carriage 1–2 months post booster.

### Case ascertainment

The details of case ascertainment are found in the published protocol [4].

### Unit of inference

The unit of inference is the individual and analyses will be conducted at the level of the individual.

### Analysis methods

#### Pneumococcal carriage and acquisition

Analysis of the effect of the PCV booster dose on the primary endpoint of cumulative VT acquisition will employ a PP cohort using data from the five NP specimens collected between 10 and 14 months of age and similarly for the secondary endpoint of VT acquisition in the six NP specimens collected between 23 and 28 months of age. Analysis will employ Cox proportional hazards regression models comparing the hazard ratio for VT acquisition in the alternative compared to standard schedule group including terms for strata in the model and using cluster-robust standard errors or the shared frailty approach to account for clustering [24]. The same approach will be used for NVT acquisition at the aforementioned time points.

Counts of serotype-specific and all VT acquisition will be compared using Poisson regression models including covariates for previous serotype-specific acquisition, strata with non-independence of events by cluster accounted for using generalised estimating equations (GEE) assuming exchangeable correlation and robust standard errors (SE). Prevalence of serotype-specific and all VT carriage will be compared at baseline, 6, 9, 18, and 24 months of age, including a term for strata and cluster identity in the models.

#### Pneumococcal immunogenicity

For the primary endpoint, we will use a PP cohort comparing VT GMCs at age 18 months in the two groups using mixed effects linear regression, including cluster as a random effect and the stratification variable as a fixed effect in the model. Separate serotype-specific models will be run with logarithmic transformed IgG levels as the outcome variable. The alternative schedule response will be deemed superior if the null hypothesis is rejected for ≥ 10/13 serotypes at the 5% level of significance.

Comparison of proportions with serotype-specific IgG concentrations ≥ 0.35 μg/ml will use log-binomial regression with GEEs assuming exchangeable correlation and robust standard errors (SE), including a covariate for strata, reporting risk ratios for the alternative hypothesis of superiority in the alternative versus standard schedule group at 1 month post booster and at age 18 months. We will also present serotype-specific GMCs and proportions with IgG concentration ≥ 0.35 μg/ml, at 1 month post primary series and 9 months of age.

For the secondary question of association between IgG and OPA levels on serotype-specific acquisition rates between 10–14 and 23–28 months of age, immunological response values will be log-transformed and presented as GMCs + / − CI. We will use Poisson regression models, including terms for strata and cluster, to examine the relationship between IgG or OPA responses and VT acquisition rate as a composite model and also in a serotype-specific manner, estimating a rate ratio for each one standard deviation increase in IgG or OPA level.

#### Yellow fever seroresponse

The primary endpoint of seroprotection 1 month after YF vaccination in separate and co-administration groups will be compared using a PP cohort. Proportions with YF neutralising antibody titres ≥ 1:8 will be calculated with exact binomial 95% CIs. The difference in proportions and a CI will be computed using a GEE model with binomial family and identity link and exchangeable correlation structure, including covariates for strata. If the lower limit of the CI for the difference in proportions is greater than − 10%, the seroresponse in the co-administration group will be deemed non-inferior.

#### Pneumococcal carriage density

Analysis of colonisation density will sum log_10_-transformed density values for single or multiple VT serotypes in each participant. The difference in VT density between groups will be estimated using linear regression models including a term for strata and GEEs to account for clustering, testing the alternative hypothesis of lower density in the alternative versus standard schedule group. Exploratory serotype-specific analyses will also be performed.

#### Presentation of results

The results of the primary endpoint analysis of pneumococcal acquisition will be presented as in Table 4. Immunogenicity results will be presented as in Table 5. Secondary endpoints will be presented as in Supplementary Tables 3, 4, and 5.

**Table 4:** Nasopharyngeal acquisition of pneumococcal serotypes at 10–14 months of age by group

Serotype	Alternative schedule *N* participants		Standard schedule *N* participants		Incidence rate ratio (95% CI)
	No. event	% (95% CI)	No. event	% (95% CI)	
1	*n*	% (95% CI)	*n*	% (95% CI)	*x.y* (95% CI)
3	*n*	% (95% CI)	*n*	% (95% CI)	*x.y* (95% CI)
4	*n*	% (95% CI)	*n*	% (95% CI)	*x.y* (95% CI)
5	*n*	% (95% CI)	*n*	% (95% CI)	*x.y* (95% CI)
6A	*n*	% (95% CI)	*n*	% (95% CI)	*x.y* (95% CI)
6B	*n*	% (95% CI)	*n*	% (95% CI)	*x.y* (95% CI)
7F	*n*	% (95% CI)	*n*	% (95% CI)	*x.y* (95% CI)
9 V	*n*	% (95% CI)	*n*	% (95% CI)	*x.y* (95% CI)
14	*n*	% (95% CI)	*n*	% (95% CI)	*x.y* (95% CI)
18C	*n*	% (95% CI)	*n*	% (95% CI)	*x.y* (95% CI)
19A	*n*	% (95% CI)	*n*	% (95% CI)	*x.y* (95% CI)
19F	*n*	% (95% CI)	*n*	% (95% CI)	*x.y* (95% CI)
23F	*n*	% (95% CI)	*n*	% (95% CI)	*x.y* (95% CI)
6C	*n*	% (95% CI)	*n*	% (95% CI)	*x.y* (95% CI)
PCV13 VT serotypes	*n*	% (95% CI)	*n*	% (95% CI)	*x.y* (95% CI)
Non-PCV13 serotypes	*n*	% (95% CI)	*n*	% (95% CI)	*x.y* (95% CI)
All serotypes	*n*	% (95% CI)	*n*	% (95% CI)	*x.y* (95% CI)

As per the definition in Sect. 5.1.1, multiple acquisitions of the same serotype may occur

**Table 5:** Immunogenicity of an alternative versus standard schedule of PCV13 at 18 months of age

	Geometric mean concentration		Geometric mean concentration ratio (primary outcome)	Proportion of participants with IgG concentration ≥ 0.35 μg/ml		Proportions risk ratio
Serotype	Alternative schedule (*N* = *x*)	Standard schedule (*N* = *y*)	Alternative vs standard schedule	Alternative schedule (*N* = *x*)	Standard schedule (*N* = *y*)	Alternative vs standard schedule
	μg/ml (95% CI)	μg/ml (95% CI)	*x* (95% CI)	% (95% CI)	% (95% CI)	*X* (95% CI)
1						
3						
4						
5						
6A						
6B						
7F						
9 V						
14						
18C						
19A						
19F						
23F						

Alternative schedule (doses scheduled at age 6 weeks and 9 months). Standard schedule (doses scheduled at age 6, 10, and 14 weeks). Data are from a per protocol cohort

*PCV13* 13-valent pneumococcal conjugate vaccine

#### Adjustment for covariates

As noted earlier, the stratifying covariate used in the randomisation of clusters, ‘high-low cluster baseline incidence of clinical pneumonia’ will be included in all statistical models. The other variables used to generate the restricted randomisation lists will not be included in statistical models [25].

#### Effect modification

We do not have a priori interest to investigate any potential effect modifiers.

#### Assumptions to be checked for statistical methods

We will conduct a Poisson dispersion test the check our distributional assumption for the acquisition count data. We will use robust and frequently used methods for pneumococcal IgG, OPA, and YF seroresponse data without explicit checks of distributional assumptions. We will visually and statistically test log_10_-transformed carriage density data for normality of the distribution.

#### Alternative methods if distributional assumptions do not hold

If distributional assumptions or model convergence are problematic, we will evaluate alternative approaches. For pneumococcal immunogenicity, if the mixed effects model does not converge, a linear regression GEE model will be used. The primary analysis method for comparing proportions (estimating risk ratios) is log-binomial regression using GEE with exchangeable correlation and robust SEs. In the case of non-convergence, we will consider changing from a log-binomial model to a Poisson GEE model or a logistic GEE model (followed by marginal standardisation to obtain risk ratios), all with exchangeable correlation structures and robust SEs. If these models do not converge, an independent correlation structure will be assumed instead of the exchangeable correlation. For YF seroresponse, a binomial GEE model with identity link will be used. If this model does not converge, we will use Poisson GEE, logistic regression, log Poisson regression or linear regression GEE [26]. In the case of non-convergence, we may also consider performing analyses unadjusted for the stratification variable.

If the acquisition count data do not follow a Poisson distribution, we will assess the negative binomial distribution. If log_10_-transformed carriage density data are not normal, we will evaluate alternative transformations to achieve normally distributed data or use quantile regression for comparison of medians.

#### Sensitivity analyses

No sensitivity analyses are planned.

#### Sub-group analyses

We do not plan any sub-group analyses.

### Missing data

Missing data will be reported in tables and figures. Specific missing data will include NP pneumococcal carriage values between 10–14 and 23–28 months of age, pneumococcal immunogenicity values at 18 months of age, and YF seroresponse values. We do not expect missingness for these variables to be greater than 5%. Analyses will be performed separately for per protocol and intention to treat cohorts.

### Additional analyses

We do not plan any additional analyses.

### Safety

PCV13 and YF vaccine are licenced products with excellent safety profiles. Safety is being monitored in the parent PVS trial measuring the incidence of clinical events in each group [13]. Serious adverse events (SAE), which will primarily be events of hospitalisation and deaths at home detected by the BHDSS, will be reported in each group. Radiological pneumonia and VT IPD will be deemed SAEs of special interest and reported in each group.

### Statistical software

STATA version 17 or higher (College Station, TX, USA) and R software will be used for analyses.

### References

#### Non-standard statistical methods

Not applicable.

#### Data management plan

The data management plan is available upon request.

#### Trial master file

The Trial Master File is located at the MRCG at LSHTM Basse Field Station. Documents from the file are available upon request.

#### Standard operating procedures and study specific procedures

These documents are available upon request.

### Supplementary Information

**Supplementary Material 1.**

## Data Availability

The principal investigator and trial statistician will have access to the final trial dataset. Datasets will be available from the principal investigator, via application to the MRCG Scientific Coordinating Committee and GG/MRCG Joint Ethics Committee.

## Abbreviations

BHDSS: Basse Health and Demographic Surveillance System
DMC: Data Monitoring Committee
EPI: Expanded Programme on Immunisation
GG/MRCG JEC: Gambia Government/Medical Research Council Unit The Gambia Joint Ethics Committee
ICC: Intra-cluster correlation coefficient
ITT: Intention-to-treat
IPD: Invasive pneumococcal disease
LSHTM: London School of Hygiene & Tropical Medicine
MRCG: Medical Research Council Unit The Gambia
NP: Nasopharyngeal
NVT: Non-vaccine type
PCV: Pneumococcal conjugate vaccine
PP: Per-protocol
PVS: Pneumococcal vaccine schedules trial
Spn: *Streptococcus pneumoniae*
SAP: Statistical analysis plan
SE: Standard error
TSC: Trial steering committee
VT: Vaccine-type
