# Supplementary material for: Pneumococcal conjugate vaccination schedules in infants—acquisition, immunogenicity, and pneumococcal conjugate and yellow fever vaccine co-administration study: statistical analysis plan
Source: Trials. 2024 Mar 26;25:216. doi: 10.1186/s13063-024-08036-6 (PMC10964629; doi:10.1186/s13063-024-08036-6)
Supplement: Supplementary file 1 — Supplementary Material 1. [file 13063_2024_8036_MOESM1_ESM.docx]

**Pneumococcal conjugate vaccination schedules in infants – acquisition, immunogenicity, and pneumococcal conjugate and yellow-fever vaccine co-administration study**

## Statistical Analysis Plan

**Supplementary material**

**Supplementary table 1. Baseline characteristics of participants enrolled to measure immunogenicity endpoints**

| **Characteristics** | **PCV Schedule Group** | | |
| --- | --- | --- | --- |
|  | **Alternative YF/PCV separate administration** | **Alternative YF/PCV co-administration** | **Standard** |
| No. enrolled | n=x | n=z | n=y |
| Age at enrolment (days), n | n=x | n=z | n=y |
| median (IQR) | x (x-x) | z (z-z) | y (y-y) |
| Sex, n | n=x | n=z | n=y |
| female, n (%) | x (x.x%) | z (z.z%) | y (y.y%) |
| Mother's age (years), n | n=x | n=z | n=y |
| median (IQR) | x (x-x) | z (z-z) | y (y-y) |
| Gestational age at birth, n | n=x | n=z | n=y |
| median (IQR) | x (x-x) | z (z-z) | y (y-y) |
| Birth weight, n | n=x | n=z | n=y |
| median (IQR) | x (x-x) | z (z-z) | y (y-y) |
| Breast fed at enrolment, n | n=x | n=z | n=y |
| yes, n (%) | x (x.x%) | z (z.z%) | y (y.y%) |
| Age at first PCV dose (days), n | n=x | n=z | n=y |
| median (IQR) | x (x-x) | z (z-z) | y (y-y) |
| Age at second PCV dose (days), n | n=x | n=z | n=y |
| median (IQR) | x (x-x) | z (z-z) | y (y-y) |
| Age at third PCV dose (days), n | n=x | n=z | n=y |
| median (IQR) | x (x-x) | z (z-z) | y (y-y) |
| Age at PCV booster (alternative schedule) or measles vaccine (standard schedule) [days], n | n=x | n=z | n=y |
| median (IQR) | x (x-x) | z (z-z) | y (y-y) |
| Antibiotics since birth, n | n=x | n=z | n=y |
| yes, n (%) | x (x.x%) | z (z.z%) | y (y.y%) |
| Smoker in house, n | n=x | n=z | n=y |
| yes, n (%) | x (x.x%) | z (z.z%) | y (y.y%) |
| Household cooking fuel, n | n=x | n=z | n=y |
| wood, n (%) | x (x.x%) | z (z.z%) | y (y.y%) |
| charcoal, n (%) | x (x.x%) | z (z.z%) | y (y.y%) |
| gas, n (%) | x (x.x%) | z (z.z%) | y (y.y%) |
| electricity, n (%) | x (x.x%) | z (z.z%) | y (y.y%) |
| dung, n (%) | x (x.x%) | z (z.z%) | y (y.y%) |
| Infant in cooking area daily, n | n=x | n=z | n=y |
| yes, n (%) | x (x.x%) | z (z.z%) | y (y.y%) |
| No. household children aged <15 years, n | n=x | n=z | n=y |
| median (IQR) | x (x-x) | z (z-z) | y (y-y) |
| Age at 18-month blood specimen (days), n | n=x | n=z | n=y |
| median (IQR) | x (x-x) | z (z-z) | y (y-y) |
| Age at post booster/post YF vaccine blood specimen (days), n | n=x | n=z | n=y |
| median (IQR) | x (x-x) | z (z-z) | y (y-y) |

**Supplementary table 2. Cluster-level baseline characteristics of participants**

| **Characteristics** | **Group** | |
| --- | --- | --- |
| **Cluster-level** | **X** | **Y** |
| No. enrolled | n=x | n=y |
| Age at enrolment (days), n | n=x | n=y |
| Median of cluster medians (IQR) | x (x-x) | y (y-y) |
| Min-max cluster median | x-x | y-y |
| Sex, n | n=x | n=y |
| Median percentage female cluster medians (IQR) | X (x-x) | Y (y-y) |
| Min-max cluster percentage | x-x | y-y |
| Mother's age (years), n | n=x | n=y |
| Median of cluster medians (IQR) | x (x-x) | y (y-y) |
| Min-max cluster median | x-x | y-y |
| Gestational age at birth, n | n=x | n=y |
| Median of cluster medians (IQR) | x (x-x) | y (y-y) |
| Min-max cluster median | x-x | y-y |
| Birth weight, n | n=x | n=y |
| Median of cluster medians (IQR) | x (x-x) | y (y-y) |
| Min-max cluster median | x-x | y-y |
| Breast fed at enrolment, n | n=x | n=y |
| Median percentage yes cluster medians (IQR) | x (x.x%) | y (y.y%) |
| Min-max cluster median | x-x | y-y |
| Age at first PCV dose (days), n | n=x | n=y |
| Median of cluster medians (IQR) | x (x-x) | y (y-y) |
| Min-max cluster median | x-x | y-y |
| Age at second PCV dose (days) in standard schedule group, n | n=x | n=y |
| Median of cluster medians (IQR) | x (x-x) | y (y-y) |
| Min-max cluster median | x-x | y-y |
| Age at third PCV dose (days) in standard schedule group, n | n=x | n=y |
| Median of cluster medians (IQR) | x (x-x) | y (y-y) |
| Min-max cluster median | x-x | y-y |
| Age at PCV booster (alternative schedule) or measles vaccine (standard schedule) [days], n | n=x | n=y |
| Median of cluster medians (IQR) | x (x-x) | y (y-y) |
| Min-max cluster median | x-x | y-y |
| Antibiotics since birth, n | n=x | n=y |
| Median percentage yes cluster medians (IQR) | x (x.x%) | y (y.y%) |
| Min-max cluster median | x-x | y-y |
| Smoker in house, n | n=x | n=y |
| Median percentage yes cluster medians (IQR) | x (x.x%) | y (y.y%) |
| Min-max cluster median | x-x | y-y |
| Household cooking fuel, n | n=x | n=y |
| Median percentage wood cluster medians (IQR) | x (x.x%) | y (y.y%) |
| Min-max cluster median | x-x | y-y |
| Median percentage charcoal cluster medians (IQR) | x (x.x%) | y (y.y%) |
| Min-max cluster median | x-x | y-y |
| Median percentage gas cluster medians (IQR) | x (x.x%) | y (y.y%) |
| Min-max cluster median | x-x | y-y |
| Median percentage electricity cluster medians (IQR) | x (x.x%) | y (y.y%) |
| Min-max cluster median | x-x | y-y |
| Median percentage dung cluster medians (IQR) | x (x.x%) | y (y.y%) |
| Min-max cluster median | x-x | y-y |
| Infant in cooking area daily, n | n=x | n=y |
| Median percentage yes cluster medians (IQR) | x (x.x%) | y (y.y%) |
| Min-max cluster median | x-x | y-y |
| No. household children aged <15 years, n | n=x | n=y |
| Median of cluster medians (IQR) | x (x-x) | y (y-y) |
| Min-max cluster median | x-x | y-y |
| Age at first NP specimen post PCV booster (alternative schedule)  or post measles vaccine (standard schedule) [days], n | n=x | n=y |
| Median of cluster medians (IQR) | x (x-x) | y (y-y) |
| Min-max cluster median | x-x | y-y |
| No. NP specimens from 10-14 months of age, n | n=x | n=y |
| Median of cluster medians (IQR) | x (x-x) | y (y-y) |
| Min-max cluster median | x-x | y-y |
| Age at 18 month blood specimen (days), n | n=x | n=y |
| Median of cluster medians (IQR) | x (x-x) | y (y-y) |
| Min-max cluster median | x-x | y-y |
| Age at post booster/post YF vaccine blood specimen (days), n | n=x | n=y |
| Median of cluster medians (IQR) | x (x-x) | y (y-y) |
| Min-max cluster median | x-x | y-y |

**Supplementary table 3. Nasopharyngeal acquisition of non-vaccine pneumococcal serotypes at 10-14 months of age, by group**

| Serotype | Alternative schedule  N participants | | Standard schedule  N participants | | Incidence rate ratio (95% CI) | |
| --- | --- | --- | --- | --- | --- | --- |
|  | No. event | % (95% CI) | No. event | % (95% CI) |  | |
| A - rank 1 | n | % (95% CI) | n | % (95% CI) | x.y (95% CI) | |
| B - rank 2 | n | % (95% CI) | n | % (95% CI) | x.y (95% CI) | |
| C - rank 3 | n | % (95% CI) | n | % (95% CI) | x.y (95% CI) | |
| D - rank 4 | n | % (95% CI) | n | % (95% CI) | x.y (95% CI) | |
| E - rank 5 | n | % (95% CI) | n | % (95% CI) | x.y (95% CI) |  |
| F - rank 6 | n | % (95% CI) | n | % (95% CI) | x.y (95% CI) |  |
| G - rank 7 | n | % (95% CI) | n | % (95% CI) | x.y (95% CI) |  |
| H - rank 8 | n | % (95% CI) | n | % (95% CI) | x.y (95% CI) |  |
| Etc. - rank 9 | n | % (95% CI) | n | % (95% CI) | x.y (95% CI) |  |
| All non-PCV13 serotypes | n | % (95% CI) | n | % (95% CI) | x.y (95% CI) |  |

As per the definition in section 5.1.1, multiple acquisitions of the same serotype may occur.

**Supplementary table 4. Nasopharyngeal acquisition of pneumococcal serotypes at 23-28 months of age, by group**

| Serotype | Alternative schedule  N participants | | Standard schedule  N participants | | Incidence rate ratio (95% CI) | |
| --- | --- | --- | --- | --- | --- | --- |
|  | No. event | % (95% CI) | No. event | % (95% CI) |  | |
| 1 | n | % (95% CI) | n | % (95% CI) | x.y (95% CI) | |
| 3 | n | % (95% CI) | n | % (95% CI) | x.y (95% CI) | |
| 4 | n | % (95% CI) | n | % (95% CI) | x.y (95% CI) | |
| 5 | n | % (95% CI) | n | % (95% CI) | x.y (95% CI) | |
| 6A | n | % (95% CI) | n | % (95% CI) | x.y (95% CI) |  |
| 6B | n | % (95% CI) | n | % (95% CI) | x.y (95% CI) |  |
| 7F | n | % (95% CI) | n | % (95% CI) | x.y (95% CI) |  |
| 9V | n | % (95% CI) | n | % (95% CI) | x.y (95% CI) |  |
| 14 | n | % (95% CI) | n | % (95% CI) | x.y (95% CI) |  |
| 18C | n | % (95% CI) | n | % (95% CI) | x.y (95% CI) |  |
| 19A | n | % (95% CI) | n | % (95% CI) | x.y (95% CI) |  |
| 19F | n | % (95% CI) | n | % (95% CI) | x.y (95% CI) |  |
| 23F | n | % (95% CI) | n | % (95% CI) | x.y (95% CI) |  |
| 6C | n | % (95% CI) | n | % (95% CI) | x.y (95% CI) |  |
| PCV13 VT serotypes | n | % (95% CI) | n | % (95% CI) | x.y (95% CI) |  |
| Non-PCV13 serotypes | n | % (95% CI) | n | % (95% CI) | x.y (95% CI) |  |
| All serotypes | n | % (95% CI) | n | % (95% CI) | x.y (95% CI) |  |

As per the definition in section 5.1.1, multiple acquisitions of the same serotype may occur.

**Supplementary table 5. Nasopharyngeal acquisition of non-vaccine pneumococcal serotypes at 23-28 months of age, by group**

| Serotype | Alternative schedule  N participants | | Standard schedule  N participants | | Incidence rate ratio (95% CI) | |
| --- | --- | --- | --- | --- | --- | --- |
|  | No. event | % (95% CI) | No. event | % (95% CI) |  | |
| A - rank 1 | n | % (95% CI) | n | % (95% CI) | x.y (95% CI) | |
| B - rank 2 | n | % (95% CI) | n | % (95% CI) | x.y (95% CI) | |
| C - rank 3 | n | % (95% CI) | n | % (95% CI) | x.y (95% CI) | |
| D - rank 4 | n | % (95% CI) | n | % (95% CI) | x.y (95% CI) | |
| E - rank 5 | n | % (95% CI) | n | % (95% CI) | x.y (95% CI) |  |
| F - rank 6 | n | % (95% CI) | n | % (95% CI) | x.y (95% CI) |  |
| G - rank 7 | n | % (95% CI) | n | % (95% CI) | x.y (95% CI) |  |
| H - rank 8 | n | % (95% CI) | n | % (95% CI) | x.y (95% CI) |  |
| Etc. - rank 9 | n | % (95% CI) | n | % (95% CI) | x.y (95% CI) |  |
| All NVT serotypes | n | % (95% CI) | n | % (95% CI) | x.y (95% CI) |  |

As per the definition in section 5.1.1, multiple acquisitions of the same serotype may occur.
